# A Subset of CD4/CD8 Double-Negative T Cells Expresses HIV Proteins in Patients on Antiretroviral Therapy

**DOI:** 10.1128/JVI.01913-15

**Published:** 2016-02-11

**Authors:** Laura K. DeMaster, Xiaohe Liu, D. Jake VanBelzen, Benjamin Trinité, Lingjie Zheng, Luis M. Agosto, Stephen A. Migueles, Mark Connors, Lidia Sambucetti, David N. Levy, Alexander O. Pasternak, Una O'Doherty

**Affiliations:** a Department of Pathology and Laboratory Medicine, University of Pennsylvania School of Medicine, Philadelphia, Pennsylvania, USA; b Center for Cancer and Metabolism, SRI International, Menlo Park, California, USA; c Department of Basic Science, New York University College of Dentistry, New York, New York, USA; d Section of Infectious Diseases, Boston University School of Medicine and Boston Medical Center, Boston, Massachusetts, USA; e Laboratory of Immunoregulation, NIAID, National Institutes of Health, Bethesda, Maryland, USA; f Department of Medical Microbiology, Laboratory of Experimental Virology, Center for Infection and Immunity, Academic Medical Center, University of Amsterdam, Amsterdam, The Netherlands

## Abstract

A major goal in HIV eradication research is characterizing the reservoir cells that harbor HIV in the presence of antiretroviral therapy (ART), which reseed viremia after treatment is stopped. In general, it is assumed that the reservoir consists of CD4^+^ T cells that express no viral proteins. However, recent findings suggest that this may be an overly simplistic view and that the cells that contribute to the reservoir may be a diverse population that includes both CD4^+^ and CD4^−^ cells. In this study, we directly infected resting CD4^+^ T cells and used fluorescence-activated cell sorting (FACS) and fiber-optic array scanning technology (FAST) to identify and image cells expressing HIV Gag. We found that Gag expression from integrated proviruses occurred in resting cells that lacked surface CD4, likely resulting from Nef- and Env-mediated receptor internalization. We also extended our approach to detect cells expressing HIV proteins in patients suppressed on ART. We found evidence that rare Gag^+^ cells persist during ART and that these cells are often negative for CD4. We propose that these double-negative α/β T cells that express HIV protein may be a component of the long-lived reservoir.

**IMPORTANCE** A reservoir of infected cells persists in HIV-infected patients during antiretroviral therapy (ART) that leads to rebound of virus if treatment is stopped. In this study, we used flow cytometry and cell imaging to characterize protein expression in HIV-infected resting cells. HIV Gag protein can be directly detected in infected resting cells and occurs with simultaneous loss of CD4, consistent with the expression of additional viral proteins, such as Env and Nef. Gag^+^ CD4^−^ cells can also be detected in suppressed patients, suggesting that a subset of infected cells express proteins during ART. Understanding the regulation of viral protein expression during ART will be key to designing effective strategies to eradicate HIV reservoirs.

## INTRODUCTION

A reservoir of infected cells exists in HIV-infected patients on antiretroviral therapy (ART) that leads to rebound of viremia when ART is stopped and remains an important barrier to HIV cure (1–3). The majority of proviruses found in ART patients are hypermutated or contain large deletions that render these proviruses defective for replication (4). Proviruses carrying large deletions are generally not thought to be expressed since the viral genes *tat* and *rev*, which are required for efficient transcription and export of viral RNAs (5–11), are often missing or mutated (4, 12).

While the reservoir is frequently described as transcriptionally silent, several studies suggest that a portion of the HIV reservoir may be transcriptionally active in ART patients *in vivo* (13–15). Notably, up to 10% of cells containing HIV DNA appear to contain viral RNA that can be detected with primers to the *gag* region (16). In contrast, *tat* and *rev* multiply spliced RNA (msRNA) forms were detected at a much lower frequency (16). We have studied HIV expression in an *in vitro* model of latency that involves direct infection of primary resting CD4^+^ T cells in which viral spread is undetectable. Consistent with *in vivo* data from Kaiser et al. (16), we found that *gag* unspliced RNA (usRNA) is the predominant viral transcript in resting CD4 T cells infected *in vitro*, whereas *tat* and *rev* msRNA is present at much lower levels (17). We extended this work with the novel finding that Gag appears to be expressed in a fraction of infected resting T cells. Moreover, we found tantalizing evidence that a low frequency of cells also express Gag protein *in vivo* in patients on ART (18).

However, we must acknowledge a limitation to our previous studies (17, 18); there is a possibility that the detected Gag signal was due to binding of the Gag antibody to uninfected cells. For example, the Gag protein detected in infected cultures could represent unfused virions that were bound to an uninfected cell after release from a nearby, productively infected T cell. The *gag* usRNA detected in these cultures could similarly have been due to bound (“incoming”) virus as suggested by Saleh and others (19, 20). Furthermore, reverse transcriptase PCR (RT-PCR) assays that target the *gag* HIV RNA also detect read-through transcripts from upstream cellular promoters (21). Because of the possibility of bound virions and/or read-through transcription, the presence of *gag* usRNA signal does not necessarily reflect nascent long terminal repeat (LTR)-driven transcription in these experiments.

Our current studies further address the question of whether the Gag signal detected *in vitro* and *in vivo* represents true viral expression or an artifact. The question is important, as the possibility of viral expression in infected resting CD4^+^ T cells has implications for HIV eradication strategies. In addition, the development of reliable assays to measure baseline expression is essential for the accurate evaluation of therapies aimed at enhancing HIV protein expression in patients on ART. Thus, we considered it important to decipher if the Gag signal we detected in our original studies was an artifact of incoming virions or nonspecific staining.

We began by conducting experiments in our *in vitro* model of latency (17, 18) to better define the specificity of our Gag staining and to further characterize the Gag^+^ cells. We discovered that the Gag^+^ cells had a unique CD4^−^ CD8^−^ “double-negative” (DN) T cell phenotype, and we went on to show that similar cells exist in patient samples. Thus, Gag^+^ double-negative T cells may provide a unique phenotype for identifying infected cells that express HIV proteins.

## MATERIALS AND METHODS

### Ethics statement and patient cohort.

Normal donor peripheral blood mononuclear cells (PBMCs) were obtained through the University of Pennsylvania's Human Immunology Core. All normal donor identifiers were removed prior to transfer. Treated and untreated HIV-infected patients were recruited from the Clinical Research Center, NIH (Bethesda, MD) and signed informed-consent forms approved by the NIAID institutional review board (IRB). Additional patients were also recruited to donate at the University of Pennsylvania. The University of Pennsylvania IRB approved the recruitment and collection of patient samples at Hospital of the University of Pennsylvania and the transfer of materials from the NIH. All treated patients had been on ART for 2 to 9 years. Viral loads were undetectable (<20 to 75 copies per ml) for all patients at the time of sample collection for fiber-optic array scanning technology (FAST), RNA, and DNA assays. CD4 counts at sample collection were as follows: ART1, 624; ART2, 736 (2009), 703 (2013), and 780 (2014); ART3, 578; ART4, 869; and ART5, 1,167. Times below the limit of detection are as follows: ART1, 9 years (except one viral blip to 1,083 in 2009); ART2, 2 to 7 years; ART3, 3 years; ART4, 4 years (except two viral loads less than 100); and ART5, 4 years. Viral loads were monitored quarterly or biannually.

### Isolation of resting CD4^+^ T cells.

PBMCs from HIV-infected patients were isolated from pheresis products by Ficoll purification. CD4^+^ T cells were enriched from PBMCs by adding phycoerythrin (PE)-labeled antibodies against lineage and activation markers (CD8, CD11c, CD14, CD16, CD20, CD56, BDCA2, T cell receptor γ/δ [TCR-γ/δ], CD25, CD69, and HLA-DR), incubated with anti-PE magnetic beads, and depleted on a magnetic column to isolate a highly pure population of resting CD4^+^ T cells. Purity was determined by flow cytometry and was generally above 95%.

### Viral isolates.

NL4-3, CH058, 89.6, 89.6Δvpu, and 89.6Δnef were made by 293 transfection with a single plasmid and were provided by the University of Pennsylvania's Center for AIDS Research Viral/Molecular Core. p89.6Δvpu, p89.6Δnef, and p89.6Δenv (22, 23) were kindly provided by Ron Collman. 89.6Δenv was pseudotyped with Env_89.6_ by cotransfecting p89.6Δenv with pcDNA89.6env. (pcDNA89.6 env was obtained through the NIH AIDS Reagent Program, Division of AIDS, NIAID, NIH [catalog number 12845], and pcDNA 89.6 env was obtained from Kathleen Collins and Ronald Collman [22–24].) VRX494 (25) and VRX1090 (26) virus preparations were pseudotyped with LAI Env by transfecting 293TS with different vectors, LAI Env and VIRPAC packaging constructs (25) provided by Laurent Humeau and Nikolay Korokhov, formerly at VIRxSYS. pNL43Δ5743-7250 was made by first annealing two oligonucleotides (5′-AATTCGGCGAGGACGCGTGG-3′ and 5′-CTAGCCACGCGTCCTCGCCG-3′) to generate a staggered linker with overhanging EcoRI and NheI sites and an internal MluI site, which is underlined. pNL4-3 was digested with EcoRI and NheI, and the resulting 13,318-bp fragment was gel purified using the QIAquick gel extraction kit (Qiagen). The gel-purified pNL4-3 fragment was incubated with the linker in the presence of T4 DNA ligase (New England BioLabs) to generate pNL43Δ5743-7250, which uniquely contains an MluI site. pNL43Δ5743-7250 was screened by MluI digestion and confirmed by sequencing across the deleted region. NL43Δ5743-7250 was generated by 293 transfection with pNL43Δ5743-7250, pHXB2-env, and pCV-1. (The pHXB2-env DNA and the pCV-1 DNA were obtained through the NIH AIDS Reagent Program, Division of AIDS, NIAID, NIH; pHXB-2 was obtained from Kathleen Page and Dan Littman [27] and pCV-1 from Flossie Wong-Staal [28].) NLENG1-IRES (G1I) and NLENG1-IRES-ΔTat (G1IΔTat), i.e., NLENG1-IRES utilizing the NL4-3 backbone, have been previously described (29, 30). NLENG1-IRES-ΔTat was generated by introducing a stop codon using overlap extension PCR after the codon for amino acid 18 in Tat, between the overlapping Vpr and Rev open reading frames (ORFs), in order to inactivate Tat expression without disrupting splice junctions or expression of other HIV-1 proteins. Infectious stocks of G1I and G1IΔTat were generated by transfection into 293T cells as previously described (31).

### Virus infection.

Purified resting CD4^+^ T cells were spinoculated at 1,200 × *g* for 2 h at 25°C at a concentration of 5 × 10^6^ cells/ml in viral transfection supernatant (32). Cells were washed after spinoculation and treated with 50 μg/ml of DNase (Roche) in 10 mM MgCl_2_ for 1 h when appropriate. Cells were cultured in RPMI medium plus 20% fetal bovine serum (FBS) with 50 μM deoxynucleoside (33, 34) and 1.25 μM saquinavir or 1 μM raltegravir as indicated below. For NLENG1-IRES and NLENG1-IRESΔTat infections, in the experiment whose results are shown in Fig. 4B, cells were spinoculated at 1,200 × *g* for 2 h at 37°C at a concentration of 1.5 × 10^6^ cells per ml. Cells were then washed. Efavirenz (EFV) (1 μg/ml) was applied to cells, where indicated below, at the time of infection. Indinavir (2 μM) was applied to the cells 2 days after infection.

### Protein staining for flow cytometry and fluorescent-activated cell sorting (FACS) of cells infected *in vitro*.

Cells infected *in vitro* were harvested 3 days postinfection, and viability was determined by LIVE/DEAD aqua staining (ABi). Cells were stained with antibodies against CD3 (AF700 [BD Biosciences]) and CD4 (phycoerythrin-Cy5.5 [PE-Cy5.5; eBiosciences] or allophycocyanin [APC; Pharmingen]) and the activation markers CD25, CD69, and HLA-DR (APC [BD Biosciences]) where indicated below prior to fixation in 1% paraformaldehyde (PFA). After fixation, cells were treated with ice-cold 50% MeOH for 10 min and permeabilized with 0.1% NP-40 (35). Mouse IgG was added after permeabilization to block nonspecific binding. KC57-fluorescein isothiocyanate (FITC) was used to visualize intracellular Gag. Cell sorting experiments were performed on an ARIA instrument. For the experiment whose results are shown in Fig. 4B, intracellular staining for Gag was performed with a PE-conjugated anti-HIV-1 Gag antibody (KC57-RD1; Beckman Coulter) on cells treated with a Cytofix/Cytoperm kit (BD Biosciences). Fixation and permeabilization of cells resulted in a 1-log-unit reduction in green fluorescent protein (GFP) fluorescence (31).

### qPCR for HIV DNA.

DNA was isolated from sorted cell populations using a DNeasy isolation kit (Qiagen). Levels of integrated HIV DNA were measured by *Alu-Gag* qPCR as previously described (36). When cell numbers were insufficient to measure integrated HIV DNA (as in Fig. 5), a total HIV, RU5 quantitative PCR (qPCR) assay was substituted (36).

### RNA isolation and quantitative RT-PCR.

RNA was isolated using the method described by Boom et al. (37). cDNA synthesis was performed in a 30-μl volume per the manufacturer's instructions (ABi) with a 60-min, 50°C incubation. Primers amplifying *gag* usRNA and *tat* and *rev* msRNA have been described elsewhere (38). Primers and probe for read-through reverse transcriptase PCR (RT-PCR) were designed to target the U3 region and untranslated region (UTR) region of the HIV LTR (forward, 5′-AGTGGCGAGCCCTCAGATG-3′; reverse, 5′-CAGCAAGCCGAGTCCT-3′; and probe, 5′-CCAGAGTCACACAACAGACGGGCACA-3′).

### Fiber-optic array scanning technology (FAST).

Patient cells were thawed, centrifuged once, and resuspended in 3 ml of phosphate-buffered saline (PBS). NL4-3-infected cells were cultured for 3 days postinfection, collected, centrifuged, and resuspended in 3 ml of PBS. Approximately, 20 million patient PBMCs or NL4-3-infected CD4^+^ T cell cultures were allowed to adhere for 40 min at 37°C in 100% humidity on pretreated slides. Cells were fixed, permeabilized, and stained with KC57, mouse anti-human CD4 Alexa 647, or TCR-α/β and 4′,6-diamidino-2-phenylindole (DAPI) nuclear stain. Each slide of immunolabeled cells was scanned, and fluorescence emission from labeled cells was collected in an array of optical fibers forming a wide collection aperture. Cells that had a ratio of average wavelength intensity to target wavelength intensity greater than one were considered to be autofluorescent and were excluded by the FAST algorithm filters. Potential “hits” for Gag-expressing cells were localized to an accuracy of 40 μm by the FAST scan and then reimaged using an automated digital microscope with a 20× objective. Manual image review was performed for each positive “hit,” and debris and dye aggregates were further excluded based on morphology. To quantify the total number of PBMCs per slide, Thermo Scientific Cellomics Array VT was used to count DAPI^+^ nuclei.

## RESULTS

### Resting CD4 T cells express Gag protein after direct infection *in vitro* (Fig. 1).

We previously described Gag protein signal in our *in vitro* model of directly infected resting CD4 T cells. We were convinced of the quiescent nature of the infected cells since they lacked activation markers and included cells that are phenotypically naive (17). We interpreted our results initially to represent *de novo* protein expression (17); however, it was possible that the Gag signal originated from bound, unfused virions (19). For example, rare, activated, and productively infected cells could release virions that bind to nearby uninfected resting CD4 T cells, giving a false appearance that could be mistaken for nascent expression from a resting cell. In addition, expression from unintegrated HIV DNA has also been reported and could give rise to Gag^+^ cells (31). To address if detection of Gag represented *de novo* translation from an integrated provirus, we set out to determine if sorted Gag^+^ cells contained integrated HIV DNA. We reasoned that enrichment of integrated HIV DNA in Gag^+^ cells sorted by FACS (relative to Gag^−^ cells) would signify protein expression from integrated HIV DNA in infected cells. If, however, the Gag signal were an artifact of bound virions, we would expect Gag^+^ cells to be enriched for HIV RNA but not integrated HIV DNA.

**FIG 1:**
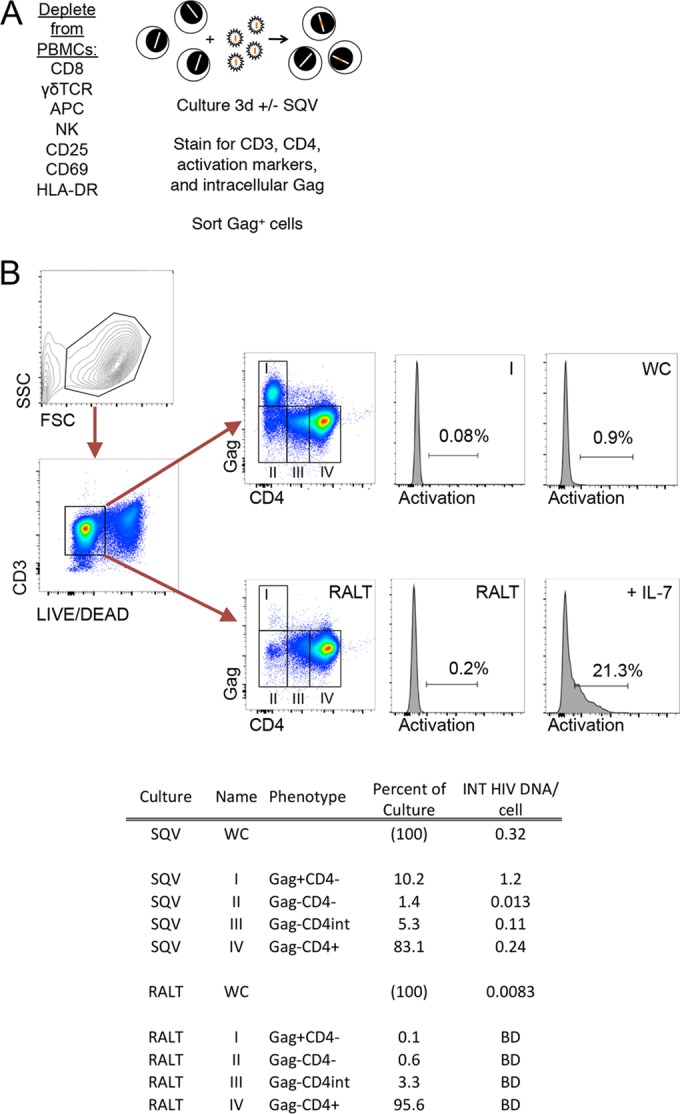
Gag expression after direct infection of resting CD4^+^ T cells is not an artifact of bound virions. (A) CD4^+^ T cells were enriched from normal donor PBMCs by negative selection and bead depletion and infected with NL4-3 by spinoculation. Cells were cultured in the absence or presence of the integrase inhibitor raltegravir for 3 days and stained for LIVE/DEAD aqua for surface markers CD3 (AF700)/CD4 (APC)/CD25/CD69/HLA-DR (PE), fixed and permeabilized, and stained with KC57 anti-Gag (PE) antibody. (B) CD3^+^ cells were gated, and cell populations I to IV were sorted. Cells cultured in the presence of raltegravir serve as a control for baseline Gag staining. Cells treated with interleukin 7 (IL-7) were also stained with CD25, CD69, and HLA-DR and serve as an activation control. The percentage of cells in each of the gates as well as the level of integrated HIV DNA in each population is summarized below the flow plots. HIV DNA was measured by *Alu*-Gag PCR in Gag^+^ cells (gate I), Gag^−^ cells (gates II to IV), and cells in the whole culture (WC). HIV integrated DNA is reported for Gag^+^ cells compared to Gag^−^ cells, showing enrichment. WC, whole culture; BD, below level of detection; SQV, saquinavir.

Quiescent CD4^+^ T cells in the G_0/1a_ stage of the life cycle were enriched by depletion of lineage and activation markers (39, 40). Cells were infected with NL4-3 by spinoculation (32) and cultured in the absence of activating cytokines and in the presence of the protease inhibitor saquinavir (17, 39). Deoxynucleosides were added to infected cultures to overcome SAMHD1-mediated restriction, which has been described to occur in resting T cells (33, 41, 42). Three days postinfection, cells were stained with LIVE/DEAD aqua and stained for surface markers with antibodies against CD3 (AF700), CD4 (PE-Cy5.5), and CD25/CD69/HLA-DR (APC). Cells were then fixed, permeabilized, and stained for HIV Gag (Fig. 1A). Surprisingly, we found that the Gag^+^ cells expressed surface CD3 but were negative for CD4 after direct infection (Fig. 1B). Consistent with our previous work, the cultured Gag^+^ cells lacked the activation markers CD25, CD69, and HLA-DR at day 3. Notably, CD4^+^ T cells cultured in the absence of antigen-presenting cells and cytokines have lower levels of TCR-ζ chain phosphorylation, suggesting that the process of culturing cells results in a lower activation state (43). The level of integrated HIV DNA was determined to be 0.32 copy per cell in the bulk culture (termed whole culture [WC]). Gag^+^ CD4^−^ cells contained 1.2 HIV proviruses per cell, which represented an enrichment of integrated HIV DNA in Gag^+^ CD4^−^ cells compared to Gag^−^ cell populations (Gag^−^ CD4^−^ cells contained 0.013 copy, Gag^−^ CD4^int^ cells contained 0.11 copy, and Gag^−^ CD4^+^ cells contained 0.24 copy of integrated HIV DNA per cell). The enrichment of integrated HIV DNA among the Gag^+^ cells suggests that the detected Gag signal reflected *de novo* protein expression and not bound virions. Furthermore, cells cultured in the presence of the integrase inhibitor raltegravir had very low Gag staining, indicating that the Gag signal was enhanced with viral integration (Fig. 1B).

### Gag^+^ CD4^−^ cells are α/β T cells (Fig. 2).

**FIG 2:**
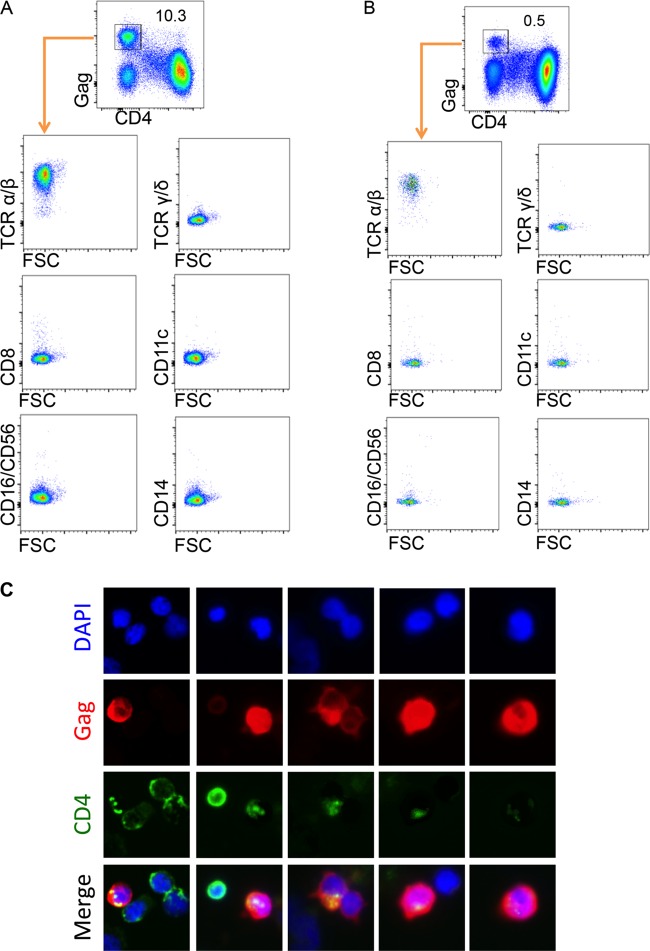
Gag^+^ CD4^−^ cells are alpha/beta T cells with internal, punctate CD4 staining. Normal donor cells were infected with either NL4-3 (A) or the R5-tropic primary isolate CHO58 (B) and stained as for Fig. 1. One PE-labeled antibody was added to each well for the following lineage markers: TCR-α/β, TCR-γ/δ, CD8, CD11c, CD16/CD56, or CD14. (C) Cells from infected cultures in panel A were plated to glass slides, fixed, and permeabilized prior to staining with antibodies against HIV Gag (red) and CD4 (green) as well as a nuclear stain (blue). Single-color and merged panels are shown to demonstrate the presence of internalized CD4 seen in Gag^+^ cells.

The infected Gag^+^ cells lacking surface CD4 (Fig. 1) warranted further investigation. To further characterize the Gag^+^ CD4^−^ T cells, we performed surface marker phenotyping. Direct infection with the X4-tropic NL4-3 was performed as for Fig. 1. The resulting infection yielded a culture in which ∼10% of cells had a Gag^+^ CD4^−^ phenotype, suggesting that surface CD4 was lost during the 3-day culture period. Gag^+^ CD4^−^ cells expressed surface T cell receptor α/β and were negative for T cell receptor γ/δ, CD8, CD11c, CD14, and CD16/CD56 (Fig. 2A). Importantly, cells infected with the primary R5-tropic isolate CHO58 (44) showed a similar phenotype (Fig. 2B). Given that the process of HIV fusion utilizes CD4, it is likely that the identified Gag^+^ CD4^−^ cells were indeed, at one time genuine CD4^+^ T cells.

To confirm the FACS observations by imaging, cells from infected cultures were plated on glass slides, and antibodies against CD4 and Gag were added after fixation and permeabilization. Fiber-optic array scanning technology (FAST) (45) was used to image cells positive for HIV Gag and CD4. Merged images of CD4 and Gag staining were generated for cells that stained positive for Gag. We identified Gag^+^ cells with punctate intracellular CD4 staining (Fig. 2C). The punctate CD4 staining pattern was unique to Gag^+^ cells. The lack of surface CD4 by FACS (Fig. 2A) and the punctate staining pattern by FAST (Fig. 2C) are consistent with internalized CD4 in Gag^+^ cells.

### Viral protein expression is responsible for CD4 internalization in infected resting cells (Fig. 3).

**FIG 3:**
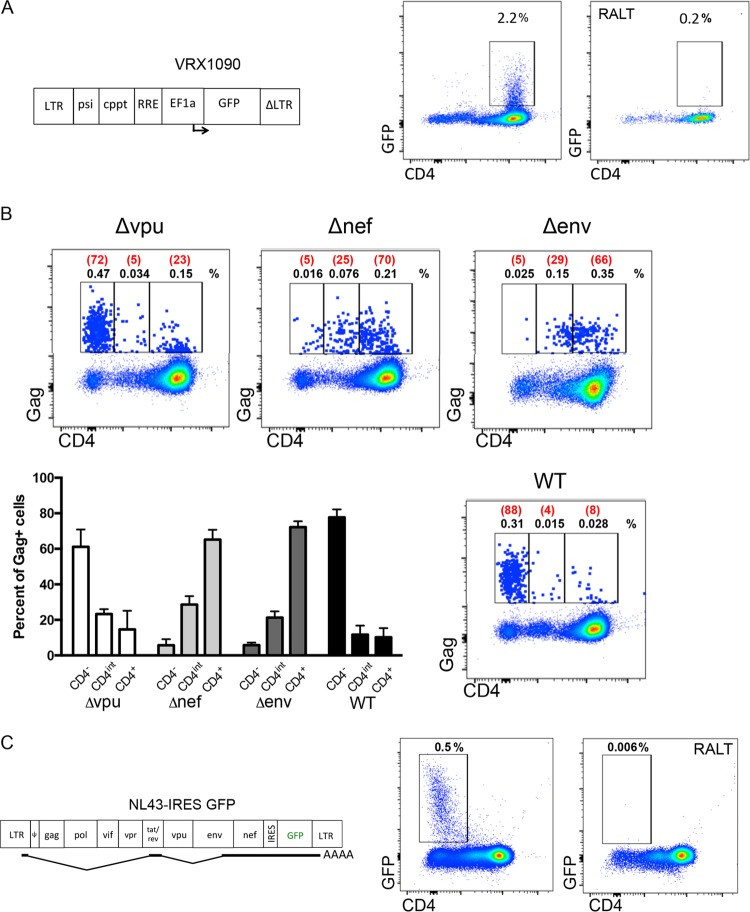
Viral protein expression is responsible for CD4 downregulation in infected resting cells. (A) VRX1090, which lacks viral genes and is engineered to express GFP under the control of the EF1a promoter, was used to infect enriched CD4 cultures. Wild-type levels of surface CD4 were observed in GFP^+^ cells, suggesting that CD4 downregulation is not due to lentiviral infection alone. (B) Cells were infected with HIV-89.6 containing defective *vpu*, *nef*, or *env* genes or wild-type 89.6 (WT) and cultured for 3 days as indicated. Surface CD4 and intracellular Gag levels were measured. The percentages of Gag^+^ CD4^−^, Gag^+^ CD4^int^ (indicating intermediate levels of CD4), and Gag^+^ CD4^+^ cells in the whole culture are shown in black. The percentages of Gag^+^ CD4^−^, Gag^+^ CD4^int^, and Gag^+^ CD4^+^ cell populations within the Gag^+^ population are shown in red. The mean percentage of each population relative to all Gag^+^ cells from three separate experiments is summarized in the bar graph. (C) CD4-enriched cultures were infected with NL43-IRES-GFP, which expresses GFP from *nef* msRNA transcripts. GFP expression and CD4 staining were evaluated at day 3.

Our results suggested that the lack of surface CD4 was related to expression of viral proteins. However, incoming virions might also induce this phenotype. For example, HIV Env on incoming virions might mask CD4 on the cell surface, or cross-linking of CD4 by HIV Env might induce CD4 internalization. To test whether infection alone could cause the CD4^−^ phenotype, we used the gutted gene therapy vector VRX1090 (26), which lacks viral genes and was engineered to express GFP driven by the EF1α promoter in infected cells (Fig. 3A). Resting CD4^+^ T cells were infected with VRX1090 that was pseudotyped with X4 HIV Env (LAI) and cultured in the absence or presence of the integrase inhibitor raltegravir, and GFP expression was evaluated on day 3 postinfection. Infected cells that expressed GFP had wild-type levels of surface CD4 (Fig. 3A), suggesting that infection alone with an HIV Env-pseudotyped virus did not lead to CD4 internalization. Moreover, these data suggested that viral gene expression is required for CD4 internalization in resting cells.

Given that multiple viral proteins (Vpu, Env, and Nef) can lower levels of surface CD4 (46–53), we strongly suspected that other viral proteins besides Gag were expressed in these infected resting T cells. To dissect which viral proteins contributed to the loss of surface CD4 in our system, we performed infections of resting cells with either wild-type 89.6 virus or 89.6 viruses lacking *vpu*, *nef*, or *env* (Fig. 3B) (22, 23). Mutations in *nef* and *env* alone resulted in intermediate levels of surface CD4, while virus carrying a mutation in the *vpu* gene showed a nearly complete loss of surface CD4, a phenotype similar to that of wild-type virus (Fig. 3B). Our experiments suggest that *de novo* synthesis of Env and Nef likely contributed to the CD4 downregulation phenotype.

We previously showed that directly infected resting CD4^+^ T cells expressed very low levels of Env (17), but we had not addressed whether Nef could be expressed in resting cells after direct infection. To determine if Nef was also expressed, we infected cultures with NL4-3-IRES-GFP, which expresses GFP from *nef* msRNA transcripts (54). We detected GFP expression in resting cells, indicating Nef expression, on day 3 postinfection (Fig. 3C). This is consistent with high levels of *nef* msRNA reported in other direct infection models (55–57). Notably, the cells expressing Nef/GFP also lacked surface CD4 (Fig. 3C).

### Defective proviruses can express low levels of HIV proteins in the absence of *tat/rev* but may not completely downregulate CD4 (Fig. 4).

**FIG 4:**
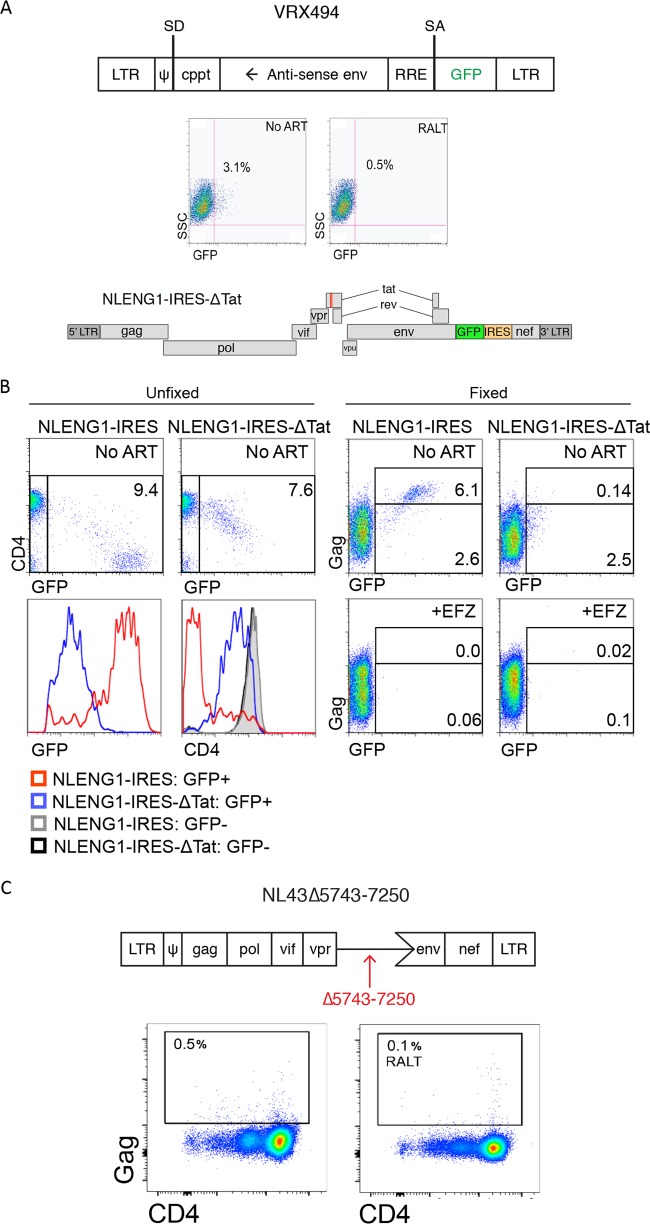
Defective proviruses may express HIV proteins in the absence of *tat* and *rev* but do not fully downregulate CD4. (A) Resting CD4-enriched cultures were infected with the gutted gene therapy vector VRX494, in which the HIV LTR drives GFP expression from a spliced message. GFP expression was measured 3 days postinfection. (B) Schematic of NLENG1-IRESΔtat, which contains a stop codon after the codon for amino acid 18 of *tat*. Cells were infected with either the parental NLENG1-IRES or NLENG1-IRESΔtat, and GFP and CD4 expression were determined at 5 days postinfection in unfixed cultures. A histogram plot is shown comparing the levels of GFP and CD4 expression in cultures infected with either NLENG1-IRES or NLENG1-IRESΔtat (left). Cells cultured in parallel were fixed, permeabilized, and stained for Gag (right). As a control, cells were infected in the presence of the reverse transcriptase inhibitor efavirenz (EFV). (C) Schematic of NL43Δ5743-7250, which lacks the *tat* and *rev* genes as well as the *env* start codon. Resting, CD4-enriched cultures were infected with NL43Δ5743-7250 and levels of surface CD4 and intracellular Gag were measured at day 3 postinfection.

Given the important roles of Tat and Rev in enhancing viral gene expression (reviewed in references 11 and 58), we were surprised by the relatively high levels of HIV Gag in the apparent absence of *tat* and *rev* msRNA in our infected resting cells (17). However, it was possible that *tat* and *rev* msRNA was present but not detected in our system; thus, we addressed whether these genes were required for LTR-driven viral gene expression by infecting resting cells with viruses lacking the *tat* and *rev* genes in our *in vitro* system. Resting CD4^+^ T cells were infected by spinoculation with Env-pseudotyped virions containing the viral vector VRX494 (25), which lacks the *tat*, *rev*, *vif*, *vpr*, *vpu*, and *nef* genes but contains the HIV LTR promoter and an ORF that encodes GFP and viral genetic elements necessary for reverse transcription and integration (Fig. 4A). Three days postinfection, a subset of infected cells (3%) expressed low levels of GFP (Fig. 4A), demonstrating that basal LTR-driven expression of viral genes was detectable in the absence of HIV accessory proteins (11, 58). This was above the background frequency of GFP^+^ cells detected in the cells cultured in the presence of raltegravir. Unintegrated HIV DNA was not expected to contribute to expression in this experiment, since Vpr is required for expression from unintegrated HIV DNA and because we assayed early after infection, before expression from unintegrated HIV DNA occurs (28). Thus, we can detect proviral gene expression in resting CD4 T cells without *tat* or *rev*.

The low-level expression of GFP in resting cells infected with lentiviral vector lacking *tat* and *rev* and all other HIV accessory proteins (Fig. 4A) raises the question of whether defective proviruses could be expressed in CD4^+^ T cells. This has clinical implications in light of the predominance of defective proviruses in HIV-infected individuals. We next asked whether proviruses with a mutation in *tat* could express viral proteins. We chose to use NLENG1-IRESΔtat, a sensitive reporter virus that contains a GFP cassette inserted upstream of the *nef* gene and a stop codon after the first 18 amino acids of Tat (Fig. 4C). Resting cells were infected with NLENG1-IRESΔtat and its parent virus, and GFP expression and CD4 levels were measured 5 days postinfection (Fig. 4B). GFP^+^ cells were detected in cultures infected with NLENG1-IRES (59) and NLENG1-IRESΔtat; however, the mode level of GFP expression was 28-fold lower in cells infected with NLENG1-IRESΔtat than with the parent NLENG1-IRES virus. Thus, Tat transactivates HIV-1 expression in resting CD4 T cells even though we failed to detected it by RT-PCR (17). Nonetheless, cells infected with NLENG1-IRESΔtat expressed low levels of viral protein, again indicating a detectable level of basal or Tat-independent transcription. Parallel infected cultures were fixed and stained for intracellular Gag (Fig. 4B). Though fixation reduced GFP fluorescence by an order of magnitude (31), Gag^+^ GFP^+^ cells were readily detected in cultures infected with the parent virus that expressed wild-type Tat (NLENG1-IRES). In addition to GFP, there is a suggestion that Gag may also be expressed with NLENG1-IRESΔtat, though the levels are very near background, suggesting that Gag expression may occur at low levels in the absence of Tat.

To determine if proviruses with large deletions in the region of *tat*, *rev*, and *env* can express detectable viral Gag in quiescent CD4 T cells, we generated a mutant NL4-3 virus with a deletion between nucleotides 5743 to 7250 (Fig. 4C). The resulting virus, NL43Δ5743-7250, was similar to proviruses reported recently by Ho et al. (4). We then infected resting CD4^+^ T cells by spinoculation using X4-tropic Env-pseudotyped NL43Δ5743-7250 virions and cultured the cells for 3 days. In three independent experiments, we consistently found very low levels of Gag just above the background levels as assessed by an integrase inhibitor, raltegravir, used as a control (Fig. 4C). Taken together, our results with sensitive reporter viruses suggest that very low-level expression of viral proteins can occur in resting cells infected with defective proviruses that contain deletions in the region of *tat*, *rev*, and *env*. However, flow assays to detect Gag expression from Tat mutants are near background (Fig. 4C) and thus distinguishable from replication-competent HIV (Fig. 1). These findings may have important implications for immune eradication strategies and methods to monitor them. Notably, low-level protein expression from defective proviruses could be visible to the primed immune system.

### FAST can identify Gag^+^ cells present at a low frequency (Fig. 5).

The FAST (fiber-optic array scanning technology) platform is an alternative method for the identification of rare cells using fluorescent detection and laser scanning technology (45). What distinguishes FAST from other cell-phenotyping techniques is its scanning speed, which enables the quantitative analysis of 20 million cells per minute. Since FAST is performed on standard microscopy slides, an automated digital microscope systematically performs fluorescent imaging on each putative rare cell and is capable of analyzing up to 6 fluorophores. Thus, all rare events can be visualized and efficiently recorded with high-resolution microscopy.

**FIG 5:**
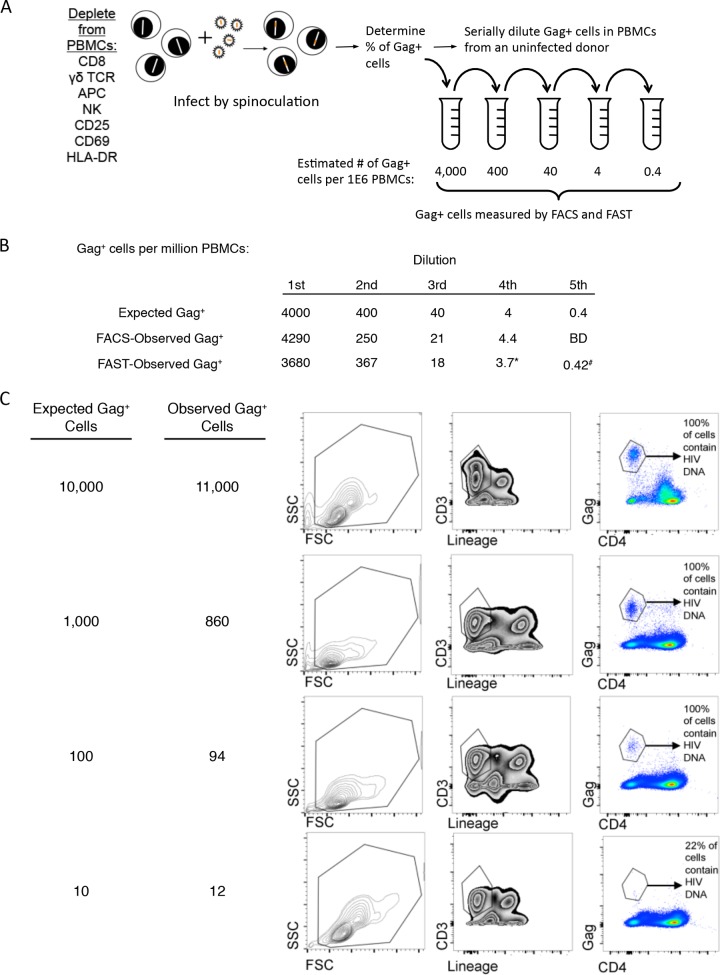
Identifying the expressed reservoir by FACS and FAST. (A) Cultures enriched for CD4 cells were infected with NL4-3 and then serially 10-fold diluted in PBMCs from an uninfected donor. (B) Serial dilutions of NL4-3-infected cells were stained for surface markers and then permeabilized and stained for Gag as for Fig. 1. The expected number of Gag^+^ cells per million PBMCs is shown in the top row at each dilution and is based on the frequency of Gag^+^ cells in the original culture as determined by flow staining; the FACS-observed number is shown in the second row and was calculated by taking the fraction identified by flow cytometry in each dilution and multiplying by 1 million; the FAST-observed number is given in the third row and was determined as follows. The same serial dilutions of infected cells were plated to slides, and Gag^+^ was identified by FAST as in Fig. 2. The number of PBMCs that adhered to slides was determined by enumerating DAPI^+^ nuclei as described in Materials and Methods. The number of Gag^+^ cells per million PBMCs is reported. *, we counted 19 Gag^+^ cells among 5.1 million PBMCs. #, we counted 3 Gag^+^ cells among 7.1 million PBMCs. BD, below detection limit; we did not provide an estimate by FACS at the final dilution because only 3 million cells were available for FACS analysis. (C) In a follow-up experiment, enriched CD4 cells from a healthy donor were infected with NL4-3 and harvested 3 days postinfection. Serial dilutions of infected cultures were made in uninfected PBMCs and stained for Gag 3 days postinfection as described for Fig. 1. Gag^+^ CD4^−^ cells were sorted, and HIV DNA was measured in Gag^+^ CD4^−^ cells by qPCR. The percentage of sorted cells that contained integrated HIV DNA is listed to show the limitation of FACS at low dilution.

Since FAST provides an independent technology for the identification of rare cells as well as image-based verification, we were interested to determine if FAST could detect HIV-infected cells that express viral proteins in a patient on ART. As a proof-of-principle experiment, resting CD4^+^ T cells were infected *in vitro* and serially diluted in a background of uninfected PBMCs to create cultures with progressively lower levels of Gag^+^ cells (Fig. 5A). In an initial experiment, both flow cytometry and FAST were used to identify and quantify Gag^+^ cells in the diluted samples. We found good agreement between flow cytometry and FAST, showing that FAST could reproducibly detect Gag^+^ cells down to a frequency of 0.4 Gag^+^ cell per million PBMCs (Fig. 5B). Notably, the dim and internalized CD4 staining shown in Fig. 2 provided additional specificity for Gag signals detected by FAST, as Gag^+^ cells generally had lower CD4 levels and often showed punctate CD4, consistent with Env-mediated or Nef-mediated CD4 internalization and degradation. To determine the accuracy of measuring the frequency of Gag^+^ cells by FACS, we made serial dilution of NL4-3-infected cultures (Fig. 5C) and measured HIV DNA in sorted Gag^+^ CD4^−^ cells. We found that FACS did not count Gag^+^ cells accurately at low frequencies. For instance, when we diluted 100 Gag^+^ CD4^−^ cells per million PBMCs, we found that 100% of sorted Gag^+^ CD4^−^ cells contained HIV DNA. However, when we diluted 10 Gag^+^ CD4^−^ cells per million PBMCs we found only 22% of the cells contained integrated HIV DNA, assuming 1 genome per cell (Fig. 5C). This is an expected limitation of flow cytometry because Gag^−^ cells are occasionally present in the same droplet as Gag^+^ cells and are sorted together, which results in a decrease of HIV DNA per sorted cell, an effect that is especially apparent at low target frequencies. Thus, FACS is a tedious, error-prone method to quantify HIV reservoirs that express proteins. On the other hand, each initial hit detected by FAST is confirmed by imaging suggesting that it would be less susceptible to spurious background events. We propose that FAST technology has the potential to provide more robust high-throughput measurements.

### FAST can be used to identify HIV-infected cells that express viral proteins in patients (Fig. 6).

**FIG 6:**
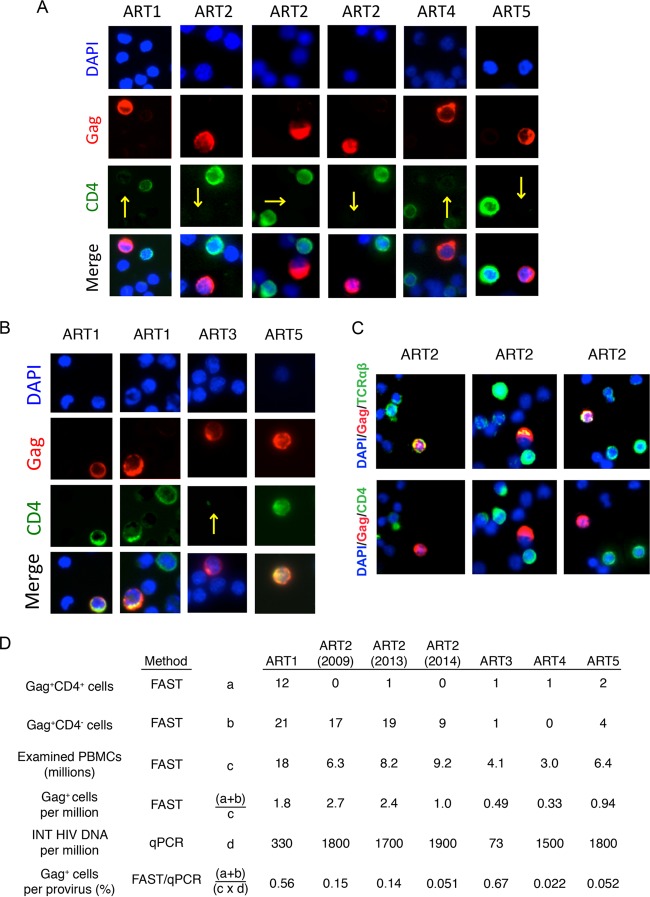
Identification of Gag^+^ cells in HIV-infected patients on ART. (A) PBMCs from ART patients were adhered to slides, fixed, permeabilized, and stained for intracellular Gag, CD4, and DNA with DAPI. Slides were scanned by FAST and imaged as for Fig. 2. Yellow arrows identify Gag^+^ CD4^−^ cells. (B) Images of Gag^+^ CD4^+^ cells and Gag^+^ CD4^int^ cells identified in ART patients. (C) PBMCs from ART2 on a replicate slide were stained for intracellular Gag, CD4, TCR-α/β, and for DNA with DAPI. Three images that contain Gag^+^ cells are shown. The top row shows staining for DAPI (blue), Gag (red), and TCR-α/β (green). The bottom row shows staining for DAPI (blue), Gag (red), and CD4 (green) for the same images. In all three images there are more TCR-α/β-positive cells than CD4^+^ T cells because of the presence of CD8^+^ T cells among the adhered PBMCs. (D) The frequencies of Gag^+^ CD4^−^ cells and Gag^+^ CD4^+^ cells were determined by FAST and divided by the total number of PBMCs. The level of integrated HIV DNA in total PBMCs was determined by *Alu*-Gag qPCR. The percentage of proviruses expressing Gag was determined by dividing the number of Gag^+^ cells per million PBMCs by the number of integrated (INT) HIV proviruses per million PBMCs and multiplying by 100.

Because each initial “hit” by FAST is confirmed by imaging, FAST is less susceptible to spurious background events that complicate the quantitation of Gag^+^ cells at low frequencies by FACS. To explore the potential of FAST, we asked if Gag^+^ cells could be identified in HIV-infected individuals on ART. PBMCs from ART patients were adhered to slides, fixed, permeabilized, and stained for CD4 and intracellular Gag. Six representative images of Gag^+^ cells that have undetectable levels of CD4 are shown (Fig. 6A). These patients had been on ART for at least 2 years and had undetectable viral loads at the time of sampling (<50 copies/ml). While the majority of Gag^+^ cells were CD4^−^, some Gag^+^ CD4^+^ cells were identified (Fig. 6B). The pattern of CD4 staining ranged from nearly wild-type levels to barely detectable and punctate (ART3).

We wanted to determine if the cells that expressed HIV Gag *in vivo* were analogous to the cells we studied *in vitro*. Thus, we tested if these cells also expressed TCR-α/β as in our *in vitro* system (Fig. 2). We stained a replicate slide from the same ART patient with antibodies against HIV Gag, CD4, and TCR-α/β. We show three cells that expressed HIV Gag that also expressed TCR-α/β while lacking CD4. In total, we found 12 Gag^+^ cells, all of which were TCR-α/β positive (Fig. 6C). Notably, Gag^+^ TCR-α/β^+^ cells were present at a frequency comparable to those in our previous studies that utilized a cell sorting approach to estimate the frequency of resting T cells that expressed HIV proteins (18). In this context, these Gag^+^ TCR-α/β^+^ cells likely represent HIV-infected T cells that express HIV proteins and thus internalize CD4.

Gag^+^ CD4^−^ cells and Gag^+^ CD4^+^ cells were counted and frequencies were determined by dividing the number of nuclei examined per slide (Fig. 6D). The percentage of proviruses expressing Gag was determined by dividing the number of Gag^+^ cells per million PBMCs by the number of integrated HIV proviruses per million PBMCs and multiplying by 100. Gag^+^ cells, which were mostly CD4^−^, persisted over a period of 5 years in one patient and were detectable in all 5 patients that were assayed. Our FAST studies with patients' cells suggest that Gag^+^ cells may contribute to the HIV reservoir and that FAST may be a useful method for the direct measurement of HIV expression.

### Read-through transcription represents only a minor component of *gag* usRNA signal.

**FIG 7:**
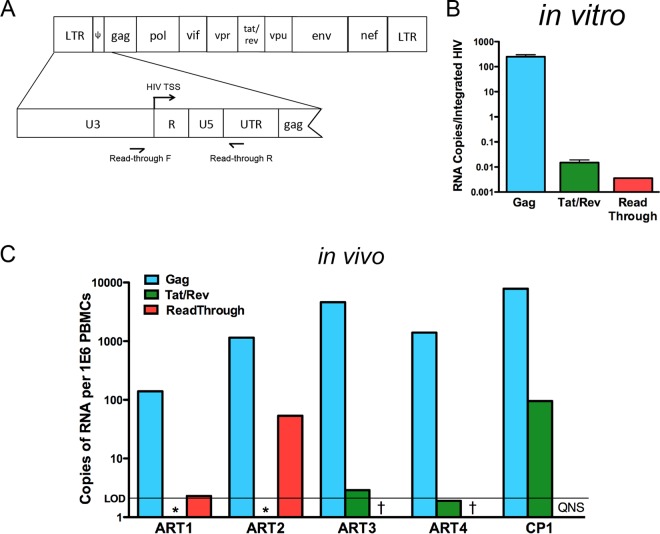
Read-through transcription represents only a minor component of *gag* usRNA signal in latently infected cells. (A) Primers were designed to amplify read-through, but not LTR-driven transcripts, by targeting U3 and UTR regions of HIV. The HIV transcriptional start site (TSS) is labeled in R. (B) RNA was isolated from NL43-infected cultures 3 days postinfection and levels of *gag* usRNA, *tat* and *rev* msRNA, and read-through RNA were quantified by RT-PCR. (C) Levels of *gag* usRNA, *tat* and *rev* msRNA, and read-through RNA were quantified in PBMCs of four ART patients and one chronic progressor (CP1). *, undetectable level of *tat* and *rev* msRNA; †, undetectable level of read-through RNA. QNS, quantity not sufficient for read-through RNA measurements.

Having demonstrated that Gag protein is expressed in resting T cells, we turned to address the composition of HIV RNA present in the infected cells. We as well as others have previously measured the levels of *gag* usRNA and *tat* and *rev* msRNA *in vitro* and *in vivo* (16, 17, 38, 60). However, we had neglected to consider the contribution of read-through transcription. Read-through transcripts initiating from an upstream cellular promoter have been reported for cells from patients on ART (21) and would be detected by primers to the *gag* region. Given that read-through transcripts should not be translated or spliced, it was important to show that a significant portion of the transcripts we detected in our system were not due to read-through. To measure the levels of read-through RNA by RT-PCR, primers and probes were designed (Fig. 7A) to bind to U3 and the 5′ untranslated region (UTR) of HIV transcripts. The forward primer targets a region upstream of the HIV transcriptional start site (TSS), such that read-through transcripts (but not *gag* usRNA transcripts) are amplified by this primer set. To compare levels of viral transcripts *in vitro*, total RNA was isolated from NL4-3-infected cultures 3 days postinfection. Read-through transcripts represented less than 0.01% of *gag* usRNA transcripts detected by RT-qPCR (Fig. 7B), suggesting that read-through transcription contributed only minimally to viral RNA expression in our *in vitro* model of direct infection of resting T cells.

We also measured levels of *gag* usRNA, *tat* and *rev* msRNA, and read-through transcripts in a total of four ART patients and one untreated chronic progressor (chronic progressor 1 [CP1]). Levels of *gag* usRNA ranged from 120 to 4,600 copies per 1E6 PBMCs in ART patients, while the chronic progressor had 7,800 copies of *gag* usRNA per 1E6 PBMCs (Fig. 7C). Read-through RNA was detected in only 2 of 4 ART patients tested (ART1 and ART2) at levels that represented less than 5% of *gag* usRNA. *tat* and *rev* msRNA was detected in ART3 and ART4 (range, 2 to 4 copies per 1E6 PBMCs) and CP1 (95 copies per 1E6 PBMCs) but not ART1 or ART2. Thus, the patterns of RNA that we detected in ART-treated patients are similar to those in our *in vitro* model. *gag* usRNA is the predominant transcript detected in ART patients and appears to be LTR driven rather than a product of read-through transcription.

## DISCUSSION

### Protein expression can occur in resting infected CD4 T cells with minimal viral spread.

Our data provide robust evidence that HIV Gag protein expression can occur in infected resting CD4 T cells. Moreover, CD4 downregulation in Gag^+^ cells suggests that these cells express multiple viral proteins. This viral protein expression occurs with minimal viral spread (17) and in cells that lack the activation markers CD25, CD69, and HLA-DR (Fig. 1). We provide evidence that the loss of surface CD4 is mediated by the Nef and Env proteins in resting cells following direct infection (Fig. 3). Tat is also expressed in these cells and is required for the high levels of viral protein expression detected (Fig. 4). The CD4^−^ phenotype and low frequency of Gag^+^ cells may explain why this protein expression has not been reported previously for patients on ART, as many studies positively select for CD4^+^ T cells before analyzing the reservoir (61).

Using our model, we show that direct infection of resting CD4^+^ T cells with wild-type viruses leads to loss of surface CD4 (Fig. 1). These Gag^+^ CD4^−^ cells express CD3 and TCR-α/β and display punctate CD4 staining when anti-CD4 antibody is added after permeabilization (Fig. 2), consistent with receptor internalization. Our previous work demonstrated that Env protein is expressed in resting CD4 cells after infection (17), and herein we provide evidence that Nef protein is also expressed in infected resting cells concurrently with the loss of surface CD4 (Fig. 3C). Notably, this finding is consistent with reports that *nef* msRNA is a prominent transcript early after direct inoculation of resting CD4^+^ T cells (55–57). Expression of both Env and Nef proteins appear to be required for complete loss of surface CD4; mutations in either gene results in partial loss of surface CD4 in Gag^+^ cells (Fig. 3B), consistent with reports of CD4 internalization in productively infected cells. Thus, both Env and Nef likely contribute to CD4 internalization, and a mutation in either gene diminishes the effect. Our data are consistent with LTR-driven expression, since read-through RNA contributes only minimally to HIV usRNA signal *in vitro* and *in vivo* (Fig. 7).

CD4 downregulation has been thought to occur only in productive infection (62, 63), but our data support the notion that CD4 downregulation can occur in infected resting cells with minimal viral replication (17). We previously showed that productive HIV infection in activated cells is associated with high levels of *tat* and *rev* msRNA, while the majority of resting cells that express HIV proteins have undetectable levels of *tat* and *rev* msRNA (17). Moreover, we easily detected spreading infection in the activated cells by many methods but could not detect viral spread in our resting T cell cultures (17). In this study, we used a sensitive reporter virus to demonstrate that Tat expression occurs in infected resting cells (Fig. 4B). Given the expression of Tat, it seems likely that these cells may release low levels of virus but to a lesser extent than has been shown for *in vitro* models with cytokine supplementation (31). In ART patients, levels of *tat* and *rev* msRNA are barely detectable (Fig. 7), and the frequency of cells with msRNA for *tat* and *rev* is much lower than the frequency of cells containing usRNA (16). Thus, rare cells that express *tat* and *rev* may be the only cells capable of releasing virions. Several groups have reported that viral RNA can be detected in the supernatant of resting cells cultured *ex vivo* (21, 64–66), consistent with the idea that very low levels of virion production may occur from resting cells. Further studies will be required to understand the extent of viral release that occurs from resting CD4^+^ T cells.

### Directly inoculated resting cells mimic many aspects of *in vivo* latent infection and provided insights into reservoir biology.

While the use of an *in vitro* model of latency is a potential limitation of our study (67), there are striking parallels between models of direct primary cell infection (68–72) and *in vivo* observations in ART patients, including the following: (i) cells infected with R5 virus have memory phenotype (17, 73–75), (ii) *gag* usRNA is the predominant viral RNA species (17, 60), (iii) low levels of *tat* and *rev* msRNA near the limit of detection of RT-PCR (16, 17), and (iv) loss of surface CD4 can be observed (Fig. 2 and 6). Importantly, it was data from our *in vitro* model that prompted us to look more aggressively for Gag^+^ CD4^−^ cells *in vivo*. Because of the strong evidence of viral protein expression provided in the resting-cell model, we used FAST to assay large numbers of cells and found the Gag^+^ CD4^−^ phenotype in five ART patients (Fig. 6). Notably, there is precedent for HIV infection and expression in double-negative T cells *in vivo*: Kaiser et al. reported an enrichment of HIV DNA and RNA in a double-negative population of CD3^+^ lymphocytes in chronic patients, but HIV DNA and RNA were not found to be enriched in CD3^+^ double-negative cells in ART patients (16). In retrospect, this study did not examine a sufficient number of cells to detect HIV nucleic acid in double-negative cells from ART patients. More recently, Yukl et al. have reported that HIV DNA is present in non-CD4 T cells in gut tissue (76). Our data raise the possibility that some of these “CD4^−^ cells” containing HIV DNA may be genuine TCR-α/β cells that have internalized or downregulated surface CD4. Images of Gag^+^ cells from ART patients demonstrate a range of CD4 staining patterns, including the canonical surface CD4 staining, punctate CD4 staining, low-level CD4 staining, and no CD4 signal (CD4^−^ cells). Thus, FAST may capture the stages of downregulation of surface CD4 in infected cells expressing HIV proteins. Studies that positively select for CD4 to study HIV reservoirs may need to be interpreted with this in mind, as this CD4^−^ population may be an important component of the reservoir.

### Defective proviruses can express viral proteins.

Using a sensitive reporter system, we showed that low-level Nef expression occurs in cells infected with a virus mutated in *tat*. Our experiments also suggest that very low protein expression may occur from proviruses containing massive 3′ deletions (Fig. 4C), similar to deletions that have been reported to occur in patients (4). Taken together, our data suggest that proviruses with large deletions may express some HIV proteins, but the expression levels are much lower in the absence of Tat (Fig. 4B and C). It follows that defective proviruses expressing viral proteins could potentially be susceptible to immune clearance. Our findings are consistent with work on retroviral vectors showing leaky expression (77, 78) and recent reports that defective proviruses can be expressed (79). We build on these reports by showing expression of defective proviruses in resting (nonactivated) cells.

### Gag^+^ cells may not be a reservoir.

Gag^+^ cells may be short-lived and not contribute to the reservoir in patients on ART. An alternate but not mutually exclusive possibility is that these protein-expressing cells may release infectious virus. Subsequently infected cells may then contribute to the persistent reservoir through low-level ongoing replication (80–82). The half-life of CD4^+^ T cells is reportedly shorter in HIV-infected patients than in uninfected controls, consistent with increased turnover of these cells in ART-treated and untreated patients (83). Finally, Gag^+^ cells may be a reservoir if low-level HIV expression occurs transiently without inducing clearance.

### A spectrum of HIV expression among reservoir cells.

Gag^+^ cells persist during ART and can be detected at multiple time points spanning 5 years (18) (Fig. 6). We hypothesize that Gag^+^ cells are long-lived and/or that viral protein expression is dynamic such that proviruses express protein transiently *in vivo*. Evidence that HIV DNA persists similarly in activated and resting cells years after ART initiation is consistent with the idea that HIV-infected cells may periodically undergo transient activation and subsequently return to a resting state (84). The role of cellular activation in reservoir expression and clearance remains to be determined in future studies, though our prior work suggests that at least a fraction of these Gag^+^ cells have a quiescent phenotype (18). We hypothesize that a spectrum of cells may exist in ART patients, including (i) cells that contain integrated provirus but do not express viral RNA, (ii) cells that contain integrated provirus and express viral RNA but not viral proteins, and (iii) cells that express both viral RNA and protein. Importantly, expression states may change over time. If Gag^+^ cells contain replication-competent provirus and are long-lived, then these cells could contribute to the reservoir. Since replication-defective proviruses may also be expressed, it may be challenging to distinguish them from replication-competent virus in HIV eradication trials (79).

### FAST may be useful to monitor reservoir expression and size.

Recent trials of HDAC inhibitors (HDACis) highlight the need for assays that detect viral protein expression. While HDACis have been shown to increase levels of HIV RNA in patient cells (66, 85), treatment with the HDACi SAHA may not induce viral protein expression in infected cells (56, 86) and thus far has not led to clearance of reservoirs *in vivo* (85, 87). The failure of SAHA to reduce reservoirs may be due to negative effects of HDACis on cytotoxic T lymphocytes (CTL) (88) or the failure of HDACis to induce protein expression (89). The fact that the reservoir persists in elite controllers who appear to have effective CTL activity against infected resting T cells expressing HIV proteins (18, 90, 91) suggests that inefficient protein expression may play a role in reservoir persistence even in the absence of ART. We speculate that the phenotype of strong Gag expression coupled with CD4 loss might be exploited to identify replication-competent virus given the very low level of Gag expressed in the absence of Tat and the requirement of high-level Nef or Env expression for complete CD4 downregulation. Thus, FAST might provide a tool to identify and possibly measure replication-competent proviruses.

## References

[1] Finzi D, Blankson J, Siliciano JD, Margolick JB, Chadwick K, Pierson T, Smith K, Lisziewicz J, Lori F, Flexner C, Quinn TC, Chaisson RE, Rosenberg E, Walker B, Gange SJ, Gallant J, Siliciano RF 1999 Latent infection of CD4+ T cells provides a mechanism for lifelong persistence of HIV-1, even in patients on effective combination therapy. Nat Med 5:512–517. doi:10.1038/8394. 10229227

[2] Chun TW, Carruth L, Finzi D, Shen X, DiGiuseppe JA, Taylor H, Hermankova M, Chadwick K, Margolick J, Quinn TC, Kuo YH, Brookmeyer R, Zeiger MA, Barditch-Crovo P, Siliciano RF 1997 Quantification of latent tissue reservoirs and total body viral load in HIV-1 infection. Nature 387:183–188. doi:10.1038/387183a0. 9144289

[3] Wong JK, Hezareh M, Günthard HF, Havlir DV, Ignacio CC, Spina CA, Richman DD 1997 Recovery of replication-competent HIV despite prolonged suppression of plasma viremia. Science 278:1291–1295. doi:10.1126/science.278.5341.1291. 9360926

[4] Ho YC, Shan L, Hosmane NN, Wang J, Laskey SB, Rosenbloom DI, Lai J, Blankson JN, Siliciano JD, Siliciano RF 2013 Replication-competent noninduced proviruses in the latent reservoir increase barrier to HIV-1 cure. Cell 155:540–551. doi:10.1016/j.cell.2013.09.020. 24243014 PMC3896327

[5] Cullen BR 1990 The HIV-1 Tat protein: an RNA sequence-specific processivity factor? Cell 63:655–657. doi:10.1016/0092-8674(90)90129-3. 2225069

[6] Sodroski JG, Rosen CA, Haseltine WA 1984 Trans-acting transcriptional activation of the long terminal repeat of human T lymphotropic viruses in infected cells. Science 225:381–421. doi:10.1126/science.6330891. 6330891

[7] Laspia MF, Rice AP, Mathews MB 1989 HIV-1 Tat protein increases transcriptional initiation and stabilizes elongation. Cell 59:283–292. doi:10.1016/0092-8674(89)90290-0. 2553266

[8] Fisher AG, Feinberg MB, Josephs SF, Harper ME, Marselle LM, Reyes G, Gonda MA, Aldovini A, Debouk C, Gallo RC, Wong-Staal F 1986 The trans-activator gene of HTLV-III is essential for virus replication. Nature 320:367–371. doi:10.1038/320367a0. 3007995

[9] Dayton AI, Sodroski JG, Rosen CA, Goh WC, Haseltine WA 1986 The trans-activator gene of the human T cell lymphotropic virus type III is required for replication. Cell 44:941–947. doi:10.1016/0092-8674(86)90017-6. 2420471

[10] Malim MH, Hauber J, Le S-Y, Maizel JV, Cullen BR 1989 The HIV-1 rev trans-activator acts through a structured target sequence to activate nuclear export of unspliced viral mRNA. Nature 338:254–257. doi:10.1038/338254a0. 2784194

[11] Karn J, Stoltzfus CM 2012 Transcriptional and posttranscriptional regulation of HIV-1 gene expression. Cold Spring Harb Perspect Med 2:a006916. 22355797 10.1101/cshperspect.a006916 PMC3281586

[12] Yukl S, Pillai S, Li P, Chang K, Pasutti W, Ahlgren C, Havlir D, Strain M, Gunthard H, Richman D, Rice AP, Daar E, Little S, Wong JK 2009 Latently-infected CD4+ T cells are enriched for HIV-1 Tat variants with impaired transactivation activity. Virology 387:98–108. doi:10.1016/j.virol.2009.01.013. 19268337 PMC4474533

[13] Furtado MR, Callaway DS, Phair JP, Kunstman BS, Stanton JL, Macken CA, Perelson AS, Wolinsky SM 1999 Persistence of HIV-1 transcription in peripheral blood mononuclear cells in patients receiving potent antiretroviral therapy. N Engl J Med 340:1614–1622. doi:10.1056/NEJM199905273402102. 10341273

[14] Lewin SR, Vesana M, Kostrikis L 1999 Use of real-time PCR and molecular beacons to detect virus replication in human immunodeficiency virus type 1-infected individuals on prolonged effective antiretroviral therapy. J Virol 73:6099–6103. 10364365 10.1128/jvi.73.7.6099-6103.1999 PMC112674

[15] Patterson BK, McCallister S, Schutz M, Siegel JN, Shults K, Flener Z, Landay A 2001 Persistence of intracellular HIV-1 mRNA correlates with HIV-1-specific immune responses in infected subjects on stable HAART. AIDS 15:1635–1641. doi:10.1097/00002030-200109070-00005. 11546937

[16] Kaiser P, Joos B, Niederost B, Weber R, Gunthard HF, Fischer M 2007 Productive human immunodeficiency virus type 1 infection in peripheral blood predominantly takes place in CD4/CD8 double-negative T lymphocytes. J Virol 81:9693–9706. doi:10.1128/JVI.00492-07. 17609262 PMC2045436

[17] Pace MJ, Graf EH, Agosto LM, Mexas AM, Male F, Brady T, Bushman FD, O'Doherty U 2012 Directly infected resting CD4+ T cells can produce HIV Gag without spreading infection in a model of HIV latency. PLoS Pathog 8:e1002818. doi:10.1371/journal.ppat.1002818. 22911005 PMC3406090

[18] Graf EH, Pace MJ, Peterson BA, Lynch LJ, Chukwulebe SB, Mexas AM, Shaheen F, Martin JN, Deeks SG, Connors M, Migueles SA, O'Doherty U 2013 Gag-positive reservoir cells are susceptible to HIV-specific cytotoxic T lymphocyte mediated clearance. PLoS One 8:e71879. doi:10.1371/journal.pone.0071879. 23951263 PMC3737195

[19] Saleh S, Wightman F, Ramanayake S, Alexander M, Kumar N, Khoury G, Pereira C, Purcell D, Cameron PU, Lewin SR 2011 Expression and reactivation of HIV in a chemokine induced model of HIV latency in primary resting CD4+ T cells. Retrovirology 8:80. doi:10.1186/1742-4690-8-80. 21992606 PMC3215964

[20] Dahabieh MS, Ooms M, Simon V, Sadowski I 2013 A doubly fluorescent HIV-1 reporter shows that the majority of integrated HIV-1 is latent shortly after infection. J Virol 87:4716–4727. doi:10.1128/JVI.03478-12. 23408629 PMC3624398

[21] Bullen CK, Laird GM, Durand CM, Siliciano JD, Siliciano RF 2014 New ex vivo approaches distinguish effective and ineffective single agents for reversing HIV-1 latency in vivo. Nat Med 20:425–429. doi:10.1038/nm.3489. 24658076 PMC3981911

[22] Collman R, Balliet JW, Gregory SA, Friedman H, Kolson DL, Nathanson N, Srinivasan A 1992 An infectious molecular clone of an unusual macrophage-tropic and highly cytopathic strain of human immunodeficiency virus type 1. J Virol 66:7517–7521. 1433527 10.1128/jvi.66.12.7517-7521.1992 PMC240461

[23] Balliet JW, Kolson DL, Eiger G, Kim FM, McGann KA, Srinivasan A, Collman R 1994 Distinct effects in primary macrophages and lymphocytes of the human immunodeficiency virus type 1 accessory genes vpr, vpu, and nef: mutational analysis of a primary HIV-1 isolate. Virology 200:623–631. doi:10.1006/viro.1994.1225. 8178448

[24] Carter CC, Onafuwa-Nuga A, McNamara LA, Riddell J, Bixby D, Savona MR, Collins KL 2010 HIV-1 infects multipotent progenitor cells causing cell death and establishing latent cellular reservoirs. Nat Med 16:446–451. doi:10.1038/nm.2109. 20208541 PMC2892382

[25] Humeau LM, Binder GK, Lu X, Slepushkin V, Merling R, Echeagaray P, Pereira M, Slepushkina T, Barnett S, Dropulic LK, Carroll R, Levine BL, June CH, Dropulic B 2004 Efficient lentiviral vector-mediated control of HIV-1 replication in CD4 lymphocytes from diverse HIV+ infected patients grouped according to CD4 count and viral load. Mol Ther 9:902–913. doi:10.1016/j.ymthe.2004.03.005. 15194057

[26] Leyva FJ, Anzinger JJ, McCoy JP Jr, Kruth HS 2011 Evaluation of transduction efficiency in macrophage colony-stimulating factor differentiated human macrophages using HIV-1 based lentiviral vectors. BMC Biotechnol 11:13. doi:10.1186/1472-6750-11-13. 21281514 PMC3045310

[27] Page KA, Landau NR, Littman DR 1990 Construction and use of a human immunodeficiency virus vector for analysis of virus infectivity. J Virol 64:5270–5276. 2214018 10.1128/jvi.64.11.5270-5276.1990 PMC248565

[28] Arya SK, Guo C, Josephs SF, Wong-Staal F 1985 Trans-activator gene of human T-lymphotropic virus type III (HTLV-III). Science 229:69–73. doi:10.1126/science.2990040. 2990040

[29] Levy DN, Aldrovandi GM, Kutsch O, Shaw GM 2004 Dynamics of HIV-1 recombination in its natural target cells. Proc Natl Acad Sci U S A 101:4204–4209. doi:10.1073/pnas.0306764101. 15010526 PMC384719

[30] Trinité B, Chan CN, Lee CS, Mahajan S, Luo Y, Muesing MA, Folkvord JM, Pham M, Connick E, Levy DN 2014 Suppression of Foxo1 activity and down-modulation of CD62L (L-selectin) in HIV-1 infected resting CD4 T cells. PLoS One 9:e110719. doi:10.1371/journal.pone.0110719. 25330112 PMC4199762

[31] Trinité B, Ohlson EC, Voznesensky I, Rana SP, Chan CN, Mahajan S, Alster J, Burke SA, Wodarz D, Levy DN 2013 An HIV-1 replication pathway utilizing reverse transcription products that fail to integrate. J Virol 87:12701–12720. doi:10.1128/JVI.01939-13. 24049167 PMC3838139

[32] O'Doherty U, Swiggard WJ, Malim MH 2000 Human immunodeficiency virus type 1 spinoculation enhances infection through virus binding. J Virol 74:10074–10080. doi:10.1128/JVI.74.21.10074-10080.2000. 11024136 PMC102046

[33] Plesa G, Dai J, Baytop C, Riley JL, June CH, O'Doherty U 2007 Addition of deoxynucleosides enhances human immunodeficiency virus type 1 integration and 2LTR formation in resting CD4+ T cells. J Virol 81:13938–13942. doi:10.1128/JVI.01745-07. 17928354 PMC2168855

[34] Korin YD, Zack JA 1999 Nonproductive human immunodeficiency virus type 1 infection in nucleoside-treated G0 lymphocytes. J Virol 73:6526–6532. 10400748 10.1128/jvi.73.8.6526-6532.1999 PMC112735

[35] Yang H, Yorke E, Hancock G, Clutton G, Sande N, Angus B, Smyth R, Mak J, Dorrell L 2013 Improved quantification of HIV-1-infected CD4+ T cells using an optimised method of intracellular HIV-1 gag p24 antigen detection. J Immunol Methods 391:174–178. doi:10.1016/j.jim.2013.03.001. 23500782

[36] Liszewski MK, Yu JJ, O'Doherty U 2009 Detecting HIV-1 integration by repetitive-sampling Alu-gag PCR. Methods 47:254–260. doi:10.1016/j.ymeth.2009.01.002. 19195495 PMC2862469

[37] Boom R, Sol CJ, Salimans MM, Jansen CL, Wertheim-van Dillen PM, van der Noordaa J 1990 Rapid and simple method for purification of nucleic acids. J Clin Microbiol 28:495–503. 1691208 10.1128/jcm.28.3.495-503.1990 PMC269651

[38] Pasternak AO, Adema KW, Bakker M, Jurriaans S, Berkhout B, Cornelissen M, Lukashov VV 2008 Highly sensitive methods based on seminested real-time reverse transcription-PCR for quantitation of human immunodeficiency virus type 1 unspliced and multiply spliced RNA and proviral DNA. J Clin Microbiol 46:2206–2211. doi:10.1128/JCM.00055-08. 18463204 PMC2446885

[39] Swiggard WJ, Baytop C, Yu JJ, Dai J, Li C, Schretzenmair R, Theodosopoulos T, O'Doherty U 2005 Human immunodeficiency virus type 1 can establish latent infection in resting CD4+ T cells in the absence of activating stimuli. J Virol 79:14179–14188. doi:10.1128/JVI.79.22.14179-14188.2005. 16254353 PMC1280214

[40] Korin YD, Zack JA 1998 Progression to the G1b phase of the cell cycle is required for completion of human immunodeficiency virus type 1 reverse transcription in T cells. J Virol 72:3161–3168. 9525642 10.1128/jvi.72.4.3161-3168.1998 PMC109773

[41] Baldauf HM, Pan X, Erikson E, Schmidt S, Daddacha W, Burggraf M, Schenkova K, Ambiel I, Wabnitz G, Gramberg T, Panitz S, Flory E, Landau NR, Sertel S, Rutsch F, Lasitschka F, Kim B, Konig R, Fackler OT, Keppler OT 2012 SAMHD1 restricts HIV-1 infection in resting CD4(+) T cells. Nat Med 18:1682–1687. doi:10.1038/nm.2964. 22972397 PMC3828732

[42] Lahouassa H, Daddacha W, Hofmann H, Ayinde D, Logue EC, Dragin L, Bloch N, Maudet C, Bertrand M, Gramberg T, Pancino G, Priet S, Canard B, Laguette N, Benkirane M, Transy C, Landau NR, Kim B, Margottin-Goguet F 2012 SAMHD1 restricts the replication of human immunodeficiency virus type 1 by depleting the intracellular pool of deoxynucleoside triphosphates. Nat Immunol 13:223–228. doi:10.1038/ni.2236. 22327569 PMC3771401

[43] Stefanová I, Dorfman JR, Germain RN 2002 Self-recognition promotes the foreign antigen sensitivity of naive T lymphocytes. Nature 420:429–434. doi:10.1038/nature01146. 12459785

[44] Ochsenbauer C, Edmonds TG, Ding H, Keele BF, Decker J, Salazar MG, Salazar-Gonzalez JF, Shattock R, Haynes BF, Shaw GM, Hahn BH, Kappes JC 2012 Generation of transmitted/founder HIV-1 infectious molecular clones and characterization of their replication capacity in CD4 T lymphocytes and monocyte-derived macrophages. J Virol 86:2715–2728. doi:10.1128/JVI.06157-11. 22190722 PMC3302286

[45] Das M, Riess JW, Frankel P, Schwartz E, Bennis R, Hsieh HB, Liu X, Ly J C, Zhou L, Nieva JJ, Wakelee HA, Bruce RH 2012 ERCC1 expression in circulating tumor cells (CTCs) using a novel detection platform correlates with progression-free survival (PFS) in patients with metastatic non-small-cell lung cancer (NSCLC) receiving platinum chemotherapy. Lung Cancer 77:421–426. doi:10.1016/j.lungcan.2012.04.005. 22555222

[46] Garcia JV, Miller AD 1991 Serine phosphorylation-independent downregulation of cell-surface CD4 by nef. Nature 350:508–511. doi:10.1038/350508a0. 2014052

[47] Rhee SS, Marsh JW 1994 Human immunodeficiency virus type 1 Nef-induced down-modulation of CD4 is due to rapid internalization and degradation of surface CD4. J Virol 68:5156–5163. 8035515 10.1128/jvi.68.8.5156-5163.1994 PMC236459

[48] Chen BK, Gandhi RT, Baltimore D 1996 CD4 down-modulation during infection of human T cells with human immunodeficiency virus type 1 involves independent activities of vpu, env, and nef. J Virol 70:6044–6053. 8709227 10.1128/jvi.70.9.6044-6053.1996 PMC190625

[49] Wildum S, Schindler M, Munch J, Kirchhoff F 2006 Contribution of Vpu, Env, and Nef to CD4 down-modulation and resistance of human immunodeficiency virus type 1-infected T cells to superinfection. J Virol 80:8047–8059. doi:10.1128/JVI.00252-06. 16873261 PMC1563805

[50] Stevenson M, Meier C, Mann AM, Chapman N, Wasiak A 1988 Envelope glycoprotein of HIV induces interference and cytolysis resistance in CD4+ cells: mechanism for persistence in AIDS. Cell 53:483–496. doi:10.1016/0092-8674(88)90168-7. 2966682 PMC9513714

[51] Geleziunas R, Bour S, Wainberg MA 1994 Cell surface down-modulation of CD4 after infection by HIV-1. FASEB J 8:593–600. 8005387 10.1096/fasebj.8.9.8005387

[52] Willey RL, Maldarelli F, Martin MA, Strebel K 1992 Human immunodeficiency virus type 1 Vpu protein induces rapid degradation of CD4. J Virol 66:7193–7200. 1433512 10.1128/jvi.66.12.7193-7200.1992 PMC240416

[53] Aiken C, Konner J, Landau NR, Lenburg ME, Trono D 1994 Nef induces CD4 endocytosis: requirement for a critical dileucine motif in the membrane-proximal CD4 cytoplasmic domain. Cell 76:853–864. doi:10.1016/0092-8674(94)90360-3. 8124721

[54] Münch J, Rajan D, Schindler M, Specht A, Rucker E, Novembre FJ, Nerrienet E, Muller-Trutwin MC, Peeters M, Hahn BH, Kirchhoff F 2007 Nef-mediated enhancement of virion infectivity and stimulation of viral replication are fundamental properties of primate lentiviruses. J Virol 81:13852–13864. doi:10.1128/JVI.00904-07. 17928336 PMC2168858

[55] Spina CA, Guatelli JC, Richman DD 1995 Establishment of a stable, inducible form of human immunodeficiency virus type 1 DNA in quiescent CD4 lymphocytes in vitro. J Virol 69:2877–2988. 10.1128/jvi.69.5.2977-2988.1995 PMC188997 7707524

[56] Mohammadi P, di Iulio J, Munoz M, Martinez R, Bartha I, Cavassini M, Thorball C, Fellay J, Beerenwinkel N, Ciuffi A, Telenti A 2014 Dynamics of HIV latency and reactivation in a primary CD4+ T cell model. PLoS Pathog 10:e1004156. doi:10.1371/journal.ppat.1004156. 24875931 PMC4038609

[57] Wu Y, Marsh JW 2001 Selective transcription and modulation of resting T cell activity by preintegrated HIV DNA. Science 293:1503–1506. doi:10.1126/science.1061548. 11520990

[58] Cullen BR, Malim MH 1990 Regulation of HIV-1 gene expression. Nucleic Acids Mol Biol 4:176–184. doi:10.1007/978-3-642-84150-7_11.

[59] Kutsch O, Benveniste EN, Shaw GM, Levy DN 2002 Direct and quantitative single-cell analysis of human immunodeficiency virus type 1 reactivation from latency. J Virol 76:8776–8786. doi:10.1128/JVI.76.17.8776-8786.2002. 12163598 PMC136999

[60] Pasternak AO, Jurriaans S, Bakker M, Prins JM, Berkhout B, Lukashov VV 2009 Cellular levels of HIV unspliced RNA from patients on combination antiretroviral therapy with undetectable plasma viremia predict the therapy outcome. PLoS One 4:e8490. doi:10.1371/journal.pone.0008490. 20046870 PMC2795168

[61] Eisele E, Siliciano RF 2012 Redefining the viral reservoirs that prevent HIV-1 eradication. Immunity 37:377–388. doi:10.1016/j.immuni.2012.08.010. 22999944 PMC3963158

[62] Piguet V, Schwartz O, Le Gall S, Trono D 1999 The downregulation of CD4 and MHC-I by primate lentiviruses: a paradigm for the modulation of cell surface receptors. Immunol Rev 168:51–63. doi:10.1111/j.1600-065X.1999.tb01282.x. 10399064

[63] Glushakova S, Munch J, Carl S, Greenough TC, Sullivan JL, Margolis L, Kirchhoff F 2001 CD4 down-modulation by human immunodeficiency virus type 1 Nef correlates with the efficiency of viral replication and with CD4(+) T-cell depletion in human lymphoid tissue ex vivo. J Virol 75:10113–10117. doi:10.1128/JVI.75.21.10113-10117.2001. 11581379 PMC114585

[64] Lassen KG, Bailey JR, Siliciano RF 2004 Analysis of human immunodeficiency virus type 1 transcriptional elongation in resting CD4+ T cells in vivo. J Virol 78:9105–9114. doi:10.1128/JVI.78.17.9105-9114.2004. 15308706 PMC506937

[65] Wei DG, Chiang V, Fyne E, Balakrishnan M, Barnes T, Graupe M, Hesselgesser J, Irrinki A, Murry JP, Stepan G, Stray KM, Tsai A, Yu H, Spindler J, Kearney M, Spina CA, McMahon D, Lalezari J, Sloan D, Mellors J, Geleziunas R, Cihlar T 2014 Histone deacetylase inhibitor romidepsin induces HIV expression in CD4 T cells from patients on suppressive antiretroviral therapy at concentrations achieved by clinical dosing. PLoS Pathog 10:e1004071. doi:10.1371/journal.ppat.1004071. 24722454 PMC3983056

[66] Cillo AR, Sobolewski MD, Bosch RJ, Fyne E, Piatak M Jr, Coffin JM, Mellors JW 2014 Quantification of HIV-1 latency reversal in resting CD4+ T cells from patients on suppressive antiretroviral therapy. Proc Natl Acad Sci U S A 111:7078–7083. doi:10.1073/pnas.1402873111. 24706775 PMC4024870

[67] Spina CA, Anderson J, Archin NM, Bosque A, Chan J, Famiglietti M, Greene WC, Kashuba A, Lewin SR, Margolis DM, Mau M, Ruelas D, Saleh S, Shirakawa K, Siliciano RF, Singhania A, Soto PC, Terry VH, Verdin E, Woelk C, Wooden S, Xing S, Planelles V 2013 An in-depth comparison of latent HIV-1 reactivation in multiple cell model systems and resting CD4+ T cells from aviremic patients. PLoS Pathog 9:e1003834. doi:10.1371/journal.ppat.1003834. 24385908 PMC3873446

[68] Cameron PU, Saleh S, Sallmann G, Solomon A, Wightman F, Evans VA, Boucher G, Haddad EK, Sekaly RP, Harman AN, Anderson JL, Jones KL, Mak J, Cunningham AL, Jaworowski A, Lewin SR 2010 Establishment of HIV-1 latency in resting CD4+ T cells depends on chemokine-induced changes in the actin cytoskeleton. Proc Natl Acad Sci U S A 107:16934–16939. 20837531 10.1073/pnas.1002894107 PMC2947912

[69] Saleh S, Solomon A, Wightman F, Xhilaga M, Cameron PU, Lewin SR 2007 CCR7 ligands CCL19 and CCL21 increase permissiveness of resting memory CD4+ T cells to HIV-1 infection: a novel model of HIV-1 latency. Blood 110:4161–4164. doi:10.1182/blood-2007-06-097907. 17881634

[70] Chavez L, Calvanese V, Verdin E 2015 HIV latency is established directly and early in both resting and activated primary CD4 T cells. PLoS Pathog 11:e1004955. doi:10.1371/journal.ppat.1004955. 26067822 PMC4466167

[71] Vatakis DN, Bristol G, Wilkinson TA, Chow SA, Zack JA 2007 Immediate activation fails to rescue efficient human immunodeficiency virus replication in quiescent CD4+ T cells. J Virol 81:3574–3582. doi:10.1128/JVI.02569-06. 17229711 PMC1866069

[72] Lucera MB, Tilton CA, Mao H, Dobrowolski C, Tabler CO, Haqqani AA, Karn J, Tilton JC 2014 The histone deacetylase inhibitor vorinostat (SAHA) increases the susceptibility of uninfected CD4+ T cells to HIV by increasing the kinetics and efficiency of postentry viral events. J Virol 88:10803–10812. doi:10.1128/JVI.00320-14. 25008921 PMC4178860

[73] Chomont N, El-Far M, Ancuta P, Trautmann L, Procopio FA, Yassine-Diab B, Boucher G, Boulassel MR, Ghattas G, Brenchley JM, Schacker TW, Hill BJ, Douek DC, Routy JP, Haddad EK, Sekaly RP 2009 HIV reservoir size and persistence are driven by T cell survival and homeostatic proliferation. Nat Med 15:893–900. doi:10.1038/nm.1972. 19543283 PMC2859814

[74] Ostrowski MA, Chun T-W, Justement SJ, Motola I, Spinelli MA, Adelsberger J, Ehler LA, Mizell SB, Hallahan CW, Fauci AS 1999 Both memory and CD45RA(+)/CD62L(+) naive CD4(+) T cells are infected in human immunodeficiency type 1-infected individuals. J Virol 73:6430–6435. 10400736 10.1128/jvi.73.8.6430-6435.1999 PMC112723

[75] Dai J, Agosto LM, Baytop C, Yu JJ, Pace MJ, Liszewski MK, O'Doherty U 2009 Human immunodeficiency virus integrates directly into naive resting CD4+ T cells but enters naive cells less efficiently than memory cells. J Virol 83:4528–4537. doi:10.1128/JVI.01910-08. 19211752 PMC2668451

[76] Yukl SA, Sinclair E, Somsouk M, Hunt PW, Epling L, Killian M, Girling V, Li P, Havlir DV, Deeks SG, Wong JK, Hatano H 2014 A comparison of methods for measuring rectal HIV levels suggests that HIV DNA resides in cells other than CD4+ T cells, including myeloid cells. AIDS 28:439–442. doi:10.1097/QAD.0000000000000166. 24322272 PMC4130176

[77] Iyer SR, Yu D, Biancotto A, Margolis LB, Wu Y 2009 Measurement of human immunodeficiency virus type 1 preintegration transcription by using Rev-dependent Rev-CEM cells reveals a sizable transcribing DNA population comparable to that from proviral templates. J Virol 83:8662–8673. doi:10.1128/JVI.00874-09. 19553325 PMC2738211

[78] Merzouki A, Patel P, Cassol S, Ennaji M, Tailor P, Turcotte FR, O'Shaughnessy M, Arella M 1995 HIV-1 gp120/160 expressing cells upregulate HIV-1 LTR directed gene expression in a cell line transfected with HIV-1 LTR-reporter gene constructs. Cell Mol Biol 41:445–452. 7580840

[79] Imamichi H, Natarajan V, Adelsberger JW, Rehm CA, Lempicki RA, Das B, Hazen A, Imamichi T, Lane HC 2014 Lifespan of effector memory CD4+ T cells determined by replication-incompetent integrated HIV-1 provirus. AIDS 28:1091–1099. doi:10.1097/QAD.0000000000000223. 24492253

[80] Chun TW, Nickle DC, Justement JS, Large D, Semerjian A, Curlin ME, O'Shea MA, Hallahan CW, Daucher M, Ward DJ, Moir S, Mullins JI, Kovacs C, Fauci AS 2005 HIV-infected individuals receiving effective antiviral therapy for extended periods of time continually replenish their viral reservoir. J Clin Invest 115:3250–3255. doi:10.1172/JCI26197. 16276421 PMC1265878

[81] Lambotte O, Chaix ML, Gubler B, Nasreddine N, Wallon C, Goujard C, Rouzioux C, Taoufik Y, Delfraissy JF 2004 The lymphocyte HIV reservoir in patients on long-term HAART is a memory of virus evolution. AIDS 18:1147–1158. doi:10.1097/00002030-200405210-00008. 15166530

[82] Coiras M, Lopez-Huertas MR, Perez-Olmeda M, Alcami J 2009 Understanding HIV-1 latency provides clues for the eradication of long-term reservoirs. Nat Rev Microbiol 7:798–812. doi:10.1038/nrmicro2223. 19834480

[83] Hellerstein M, Hanley MB, Cesar D, Siler S, Papageorgopoulos C, Wieder E, Schmidt D, Hoh R, Neese R, Macallan D, Deeks S, McCune JM 1999 Directly measured kinetics of circulating T lymphocytes in normal and HIV-1-infected humans. Nat Med 5(1):83–89. doi:10.1038/4772. 9883844

[84] Murray JM, Zaunders JJ, McBride KL, Xu Y, Bailey M, Suzuki K, Cooper DA, Emery S, Kelleher AD, Koelsch KK, Team PS 2014 HIV DNA subspecies persist in both activated and resting memory CD4+ T cells during antiretroviral therapy. J Virol 88:3516–3526. doi:10.1128/JVI.03331-13. 24403590 PMC3957951

[85] Archin NM, Liberty AL, Kashuba AD, Choudhary SK, Kuruc JD, Crooks AM, Parker DC, Anderson EM, Kearney MF, Strain MC, Richman DD, Hudgens MG, Bosch RJ, Coffin JM, Eron JJ, Hazuda DJ, Margolis DM 2012 Administration of vorinostat disrupts HIV-1 latency in patients on antiretroviral therapy. Nature 487:482–485. doi:10.1038/nature11286. 22837004 PMC3704185

[86] Lassen KG, Ramyar KX, Bailey JR, Zhou Y, Siliciano RF 2006 Nuclear retention of multiply spliced HIV-1 RNA in resting CD4+ T cells. PLoS Pathog 2:e68. doi:10.1371/journal.ppat.0020068. 16839202 PMC1487174

[87] Archin NM, Bateson R, Tripathy M, Crooks AM, Yang KH, Dahl NP, Kearney MF, Anderson EM, Coffin JM, Strain MC, Richman DD, Robertson KR, Kashuba AD, Bosch RJ, Hazuda DJ, Kuruc JD, Eron JJ, Margolis DM 2014 HIV-1 expression within resting CD4 T-cells following multiple doses of vorinostat. J Infect Dis 210:728–735. doi:10.1093/infdis/jiu155. 24620025 PMC4148603

[88] Jones RB, O'Connor R, Mueller S, Foley M, Szeto GL, Karel D, Lichterfeld M, Kovacs C, Ostrowski MA, Trocha A, Irvine DJ, Walker BD 2014 Histone deacetylase inhibitors impair the elimination of HIV-infected cells by cytotoxic T-lymphocytes. PLoS Pathog 10:e1004287. doi:10.1371/journal.ppat.1004287. 25122219 PMC4133386

[89] Shan L, Deng K, Shroff NS, Durand CM, Rabi SA, Yang H-C, Zhang H, Margolick JB, Blankson JN, Siliciano RF 2012 Stimulation of HIV-1-specific cytolytic T lymphocytes facilitates elimination of latent viral reservoir after virus reactivation. Immunity 36:491–501. doi:10.1016/j.immuni.2012.01.014. 22406268 PMC3501645

[90] Buckheit RW III, Siliciano RF, Blankson JN 2013 Primary CD8+ T cells from elite suppressors effectively eliminate non-productively HIV-1 infected resting and activated CD4+ T cells. Retrovirology 10:68. doi:10.1186/1742-4690-10-68. 23816179 PMC3702406

[91] Migueles SA, Osborne CM, Royce C, Compton AA, Joshi RP, Weeks KA, Rood JE, Berkley AM, Sacha JB, Cogliano-Shutta NA, Lloyd M, Roby G, Kwan R, McLaughlin M, Stallings S, Rehm C, O'Shea MA, Mican J, Packard BZ, Komoriya A, Palmer S, Wiegand AP, Maldarelli F, Coffin JM, Mellors JW, Hallahan CW, Follman DA, Connors M 2008 Lytic granule loading of CD8+ T cells is required for HIV-infected cell elimination associated with immune control. Immunity 29:1009–1021. doi:10.1016/j.immuni.2008.10.010. 19062316 PMC2622434

